# A red fluorescent protein with improved monomericity enables ratiometric voltage imaging with ASAP3

**DOI:** 10.1038/s41598-022-07313-1

**Published:** 2022-03-07

**Authors:** Benjamin B. Kim, Haodi Wu, Yukun A. Hao, Michael Pan, Mariya Chavarha, Yufeng Zhao, Michael Westberg, François St-Pierre, Joseph C. Wu, Michael Z. Lin

**Affiliations:** 1 Department of Bioengineering, Stanford University, Stanford, CA USA; 2 Stanford Cardiovascular Institute, Stanford University, Stanford, USA; 3 Department of Neurobiology, Stanford University, Stanford, CA USA; 4 Department of Chemistry, Aarhus University, Aarhus, Denmark; 5 Baylor College of Medicine, Houston, TX USA; 6 Present Address: Department of Medicine, Heart, Lung, Blood, and Vascular Medicine Institute, University of Pittsburgh, Pittsburgh, PA USA

**Keywords:** Fluorescence imaging, Protein design

## Abstract

A ratiometric genetically encoded voltage indicator (GEVI) would be desirable for tracking transmembrane voltage changes in the presence of sample motion. We performed combinatorial multi-site mutagenesis on a cyan-excitable red fluorescent protein to create the bright and monomeric mCyRFP3, which proved to be uniquely non-perturbing when fused to the GEVI ASAP3. The green/red ratio from ASAP3-mCyRFP3 (ASAP3-R3) reported voltage while correcting for motion artifacts, allowing the visualization of membrane voltage changes in contracting cardiomyocytes and throughout the cell cycle of motile cells.

## Introduction

Ratiometric genetically encoded voltage indicators (GEVIs) are needed to remove unwanted artifacts of membrane motion so that changes in membrane potential can be discriminated from motion-induced changes in fluorescence. Membrane motion occurs when cells migrate or change shape, for example when cultured cardiomyocytes contract. Membrane motion is also an inherent feature of contractile tissues such as skeletal muscles, the gastrointestinal tract, or the heart, and sample movement relative to the camera is an omnipresent artifact during microscopy in live animals due to breathing, hemodynamic motions, or animal movement^1^.

A properly designed ratiometric GEVI would be able to provide a readout corrected for changes in indicator abundance caused by motion if two criteria are fulfilled. First, a reference fluorophore’s brightness must be either independent or inversely correlated to that of a voltage-modulated fluorophore of different wavelengths in the same molecule, so that the ratio of emissions at the two wavelengths at any point in space can be related to voltage independently of indicator abundance at that point. Second, both the fluorophores should be excitable by the same wavelength of light, as this would enable simultaneous recording of the two fluorophores in separate channels, thereby increasing speed, simplicity, and precision. GEVIs that utilize a change in FRET efficiency between two fluorescent proteins meet these requirements, but engineering large responses in FRET-based GEVIs has proven to be difficult^2^.

Another solution may be to use a voltage-independent fluorophore with a large Stokes shift that can be excited at the same wavelength as the voltage sensitive-fluorophore while keeping their emissions easily separable. Many recently developed GEVIs with high voltage responsivity function via brightness modulation of a single fluorophore. For example, ASAP-family GEVIs utilize a single circularly permuted green fluorescent protein (GFP) domain^3–6^. We sought to create a high-performance ratiometric GEVI by fusing a voltage-independent large-Stokes-shift (LSS) red fluorescent protein (RFP) to ASAP3.

While many LSS RFPs have been developed, they suffer from residual oligomerization^7,8^ or poor brightness^9^. To create a ratiometric GEVI, we initially fused ASAP-family GEVIs at their C-terminus to the cyan-excitable red fluorescent protein CyRFP1 or its more monomeric variant mCyRFP1. The cyan excitation of CyRFP1 and mCyRFP1 allows them to be co-excited efficiently with ASAP1 by 488-nm light (or 950-nm light in two-photon mode). We also tested mRuby3, FusionRed, and mCherry, which have been fused to a variety of proteins without causing toxicity^10,11^. Disappointingly, when expressed in neurons, all five fusions accumulated in the cell body and dendrites in structures too large to represent single vesicles, suggesting protein aggregation (Supplementary Fig. 1). These accumulations were more visible in the red channel than the green channel, likely because the RFPs are located in the pH-neutral cytosol while the GFP moiety of ASAP is located in the acidic lumen of the secretory pathway. The resulting differences in green/red ratios in the cell prevents using the red channel to correct for movement artifacts. In addition, GEVI molecules trapped in intracellular accumulations will not respond to voltage changes at the membrane. As the CyRFP1 fusion appeared to be better localized to the membrane compared to other RFP fusions, we chose CyRFP1 as a template for creating a more monomeric RFP.

**Figure 1:**
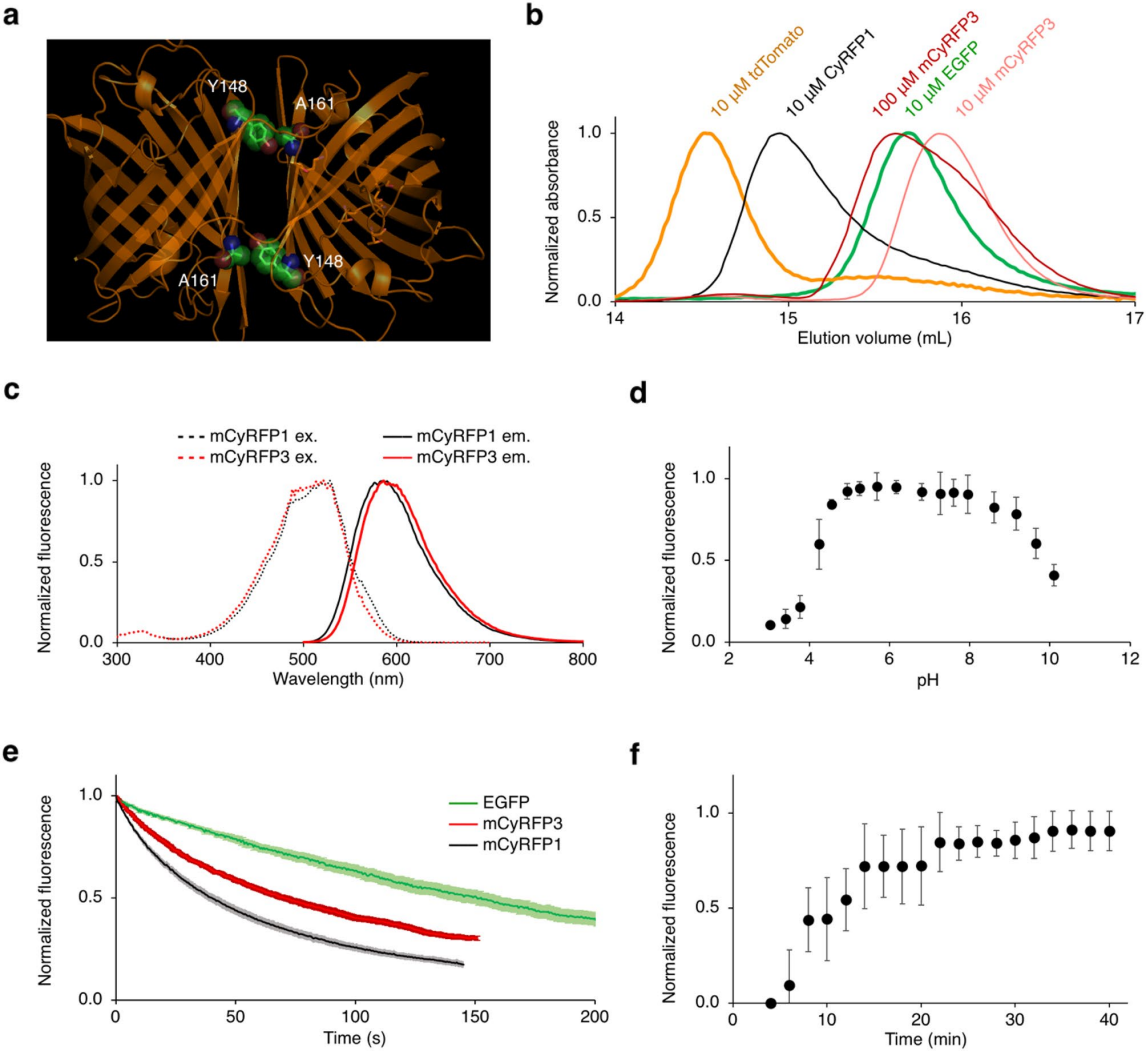
Engineering and characterization of mCyRFP3. Engineering of mCyRFP3. (**a**) Crystal structure of dimeric predecessor CyOFP1 displaying interacting residues Y148 and A161. Image was generated in Schrödinger MacPyMol 1.8. (**b**) Size-exclusion chromatography of EGFP, tdTomato, CyRFP1, and mCyRFP3. EGFP was used as a monomeric standard, while tdTomato was used as a dimeric standard. (**c**) Excitation and emission spectra of mCyRFP3 compared to its parent mCyRFP1. (**d**) pH dependence of mCyRFP3 fluorescence, demonstrating a pKa of 4.1. Error bars are s.em.m of triplicate measurements. (**e**) Photobleaching kinetics of purified cyan- excitable red fluorescent proteins under illumination by a 120-W metal-halide arc lamp through a 490/20-nm excitation filter. The time-axis was adjusted for each fluorophore to simulate excitation conditions producing 1000 photons per s per molecule. Lighter shading on CyRFP1, mCyRFP1, and mCyRFP3 lines represents standard deviation of five measurements. (**f**) Maturation kinetics of mCyRFP3 demonstrating a half-life of t = 12.5 min. Graphs in (**a**) through (**f**) were generated in Microsoft Excel for Mac 16.

mCyRFP1 had shown increased monomericity relative to CyRFP1 in vitro due to a surface A161K mutation, which breaks a hydrophobic contact with Tyr-148 across the cross-dimer interface^8^ (Fig. 1a). We hypothesized that A161K may not be the optimal mutation for breaking this hydrophobic contact, e.g. if retaining tyrosine at position 148 is not ideal for folding or hydration in the monomeric state. Indeed, performing double saturation mutagenesis and screening for brightness yielded a Y148T A161V mutant that was more monomeric in vitro (Fig. 1b). We designated this protein mCyRFP3, since the name mCyRFP2 was already assigned to a different mCyRFP1 variant^12^. mCyRFP3 exhibited absorbance and excitation centered near 500 nm and peak emission of 586 nm, slightly red-shifted from mCyRFP1 (Fig. 1c). mCyRFP3 showed improved fluorescence quantum yield of 0.72 and an extinction coefficient of 38 mM^−1^ cm^−1^ (Table 1), making its molar brightness more than 50% brighter than its predecessor mCyRFP1. mCyRFP3 fluorescence demonstrated a pKa of 4.1 and was extremely stable in the physiological pH range (Fig. 1d). mCyRFP3 photobleached with a normalized half-time of 75 s (Fig. 1e), and exhibited a maturation half-time of 12.5 min (Fig. 1f).

**Table 1:** Characteristics of mCyRFP3.

	Peak excitation (nm)^a^	Peak EC (mM^−1^ cm^−1^)	Peak emission (nm)	QY	Brightness (mM^−1^ cm^−1^)	pK_a_	Photostability (s)
CyRFP1	528 (488)	48	591	0.75	36	6.1	ND
mCyRFP1	528 (488)	27	594	0.65	17	5.8	40
mCyRFP3	522 (488)	38	588	0.72	28	4.8	70

*EC* extinction coefficient, *QY* quantum yield, *ND* not determined.

^a^A secondary peak is listed in parentheses.

**Figure 2:**
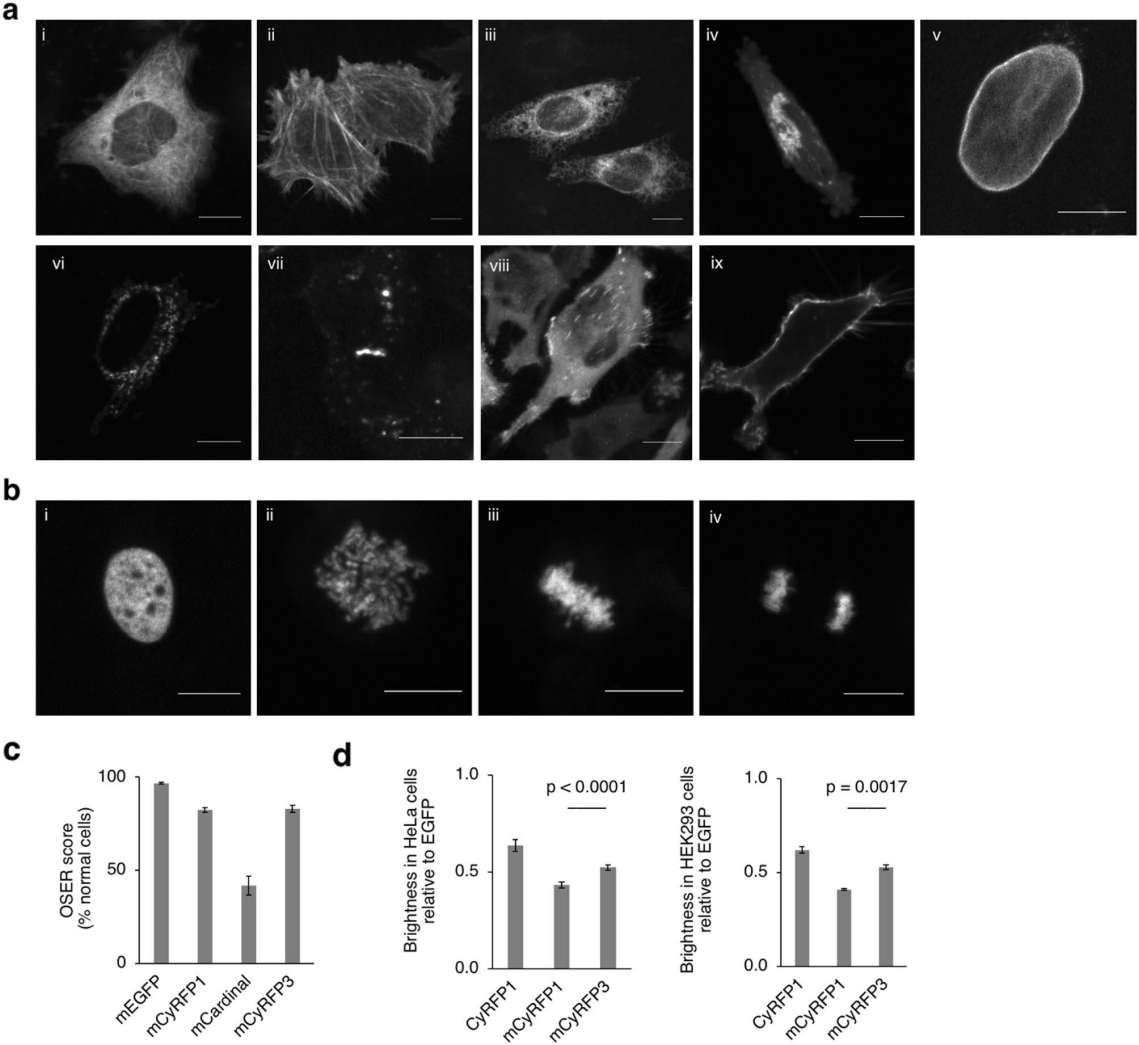
Performance of mCyRFP3 in mammalian cells. (**a**) HeLa cells expressing mCyRFP3 fused various subcellular proteins. For each fusion, the original of the fusion partner and its normal subcellular location are indicated in parentheses. (i) mCyRFP3-2aa-tubulin (human, microtubules), (ii) mCyRFP3-7aa-actin (human, actin cytoskeleton), (iii) Calnexin-14aa-mCyRFP3 (human, endoplasmic reticulum), (iv) mannosidaseII-10aa-mCyRFP3 (mouse, Golgi complex), (v) mCyRFP3-10aa-lamin B1 (human, nuclear envelope) (vi) PDHA-10aa-mCyRFP3 (human, mitochondrial pyruvate dehydrogenase), (vii) connexin43-7aa-mCyRFP3 (rat, cell–cell adhesion junctions), (viii) paxillin-22aa-mCyRFP3 (chicken, focal adhesions), (ix). mCyRFP3-2aa-CAAX. Scale bar, 10 µm. (**b**) mCyRFP3-10aa-H2B (human, nucleosomes) in (i) interphase, (ii) prophase, (iii) metaphase, (iv) anaphase. Spinning-disk confocal image stacks were acquired using Improvision Volocity 6.0 and flattened and scaled in NIH Fiji 2.1. (**c**) Performance of mCyRFP3 compared to mCyRFP1, mEGFP and mCardinal on CytERM monomericity assay. Error bars represent standard deviation of measurements from > 150 cells in each of three separate experiments. (**d**) mCyRFP3 characterization in mammalian cells. Brightness comparison of mCyRFP3 in HEK293A and HeLa cells expressing a bicistronic construct EGFP-P2A-RFP where RFP = CyRFP1, mCyRFP1, and mCyRFP3. The red fluorescence generated from each of the RFPs were normalized to the fluorescence of EGFP to normalize for expression and were charted relative to the value of CyRFP1. Excitation was performed with a 480/10-nm filter and emission was collected from 580 to 800 nm. Integrated red emission relative to the integrated GFP emission is shown as mean ± s.e.m. of 6–8 biological replicates. Graphing and two-tailed t tests with Bonferroni correction were performed in Microsoft Excel for Mac 16.

We next characterized mCyRFP3 performance in mammalian cells. Fusions to various subcellular proteins were properly localized (Fig. 2a) and a histone H2B fusion enabled observation of mitosis (Fig. 2b). In the organized smooth endoplasmic reticulum (OSER) assay, mCyRFP3 showed a similar score (87% whorl-free cells) as mCyRFP1 (Fig. 2c). While purified proteins in vitro provide extinction coefficients and quantum yield parameters as an objective measure of a mature FP’s per-molecule brightness, an FP’s effective brightness in cells is influenced by its maturation efficiency and stability. We compared the brightness of cells expressing mCyRFP3, mCyRFP1, or CyRFP1 using a bicistronic construct co-expressing EGFP, where the EGFP fluorescence was used to normalize for differences in transfection and mRNA levels. In HEK293A and HeLa cells, mCyRFP3 was significantly brighter than mCyRFP1 (Fig. 2d).

We were curious if optimization of amino acids 148 and 161 could also improve the performance of another RFP derived from eqFP578, the natural tetrameric predecessor to mCyRFP1. Thus, we also performed combinatorial saturation mutagenesis at these positions in the mMaroon1 far-red fluorescent protein, which is descended from eqFP578 via mNeptune1^13^. Indeed simultaneous Y148T A161G mutations led to the development of a more monomeric and brighter variant, mMaroon2 (Supplementary Note, Supplementary Figs. 2, 3, Supplementary Table 1).

We recently developed ASAP3, a GEVI with improved responsivity and brightness compared to previous ASAP-family GEVIs^5^. ASAP3 consists of a 4-helix transmembrane voltage-sensing domain (VSD) bearing a circularly permuted GFP domain inserted into an extracellular loop. Voltage-dependent motions of the VSD are transduced into changes in GFP fluorescence under blue light excitation. An independent comparison found that ASAP3 exhibits the largest power-normalized signal-to-noise ratios for both subthreshold voltage dynamics and action potentials (APs) among GEVIs developed to date^14^. We thus explored whether mCyRFP3 could serve as a non-perturbing voltage-independent label for ASAP3.

**Figure 3:**
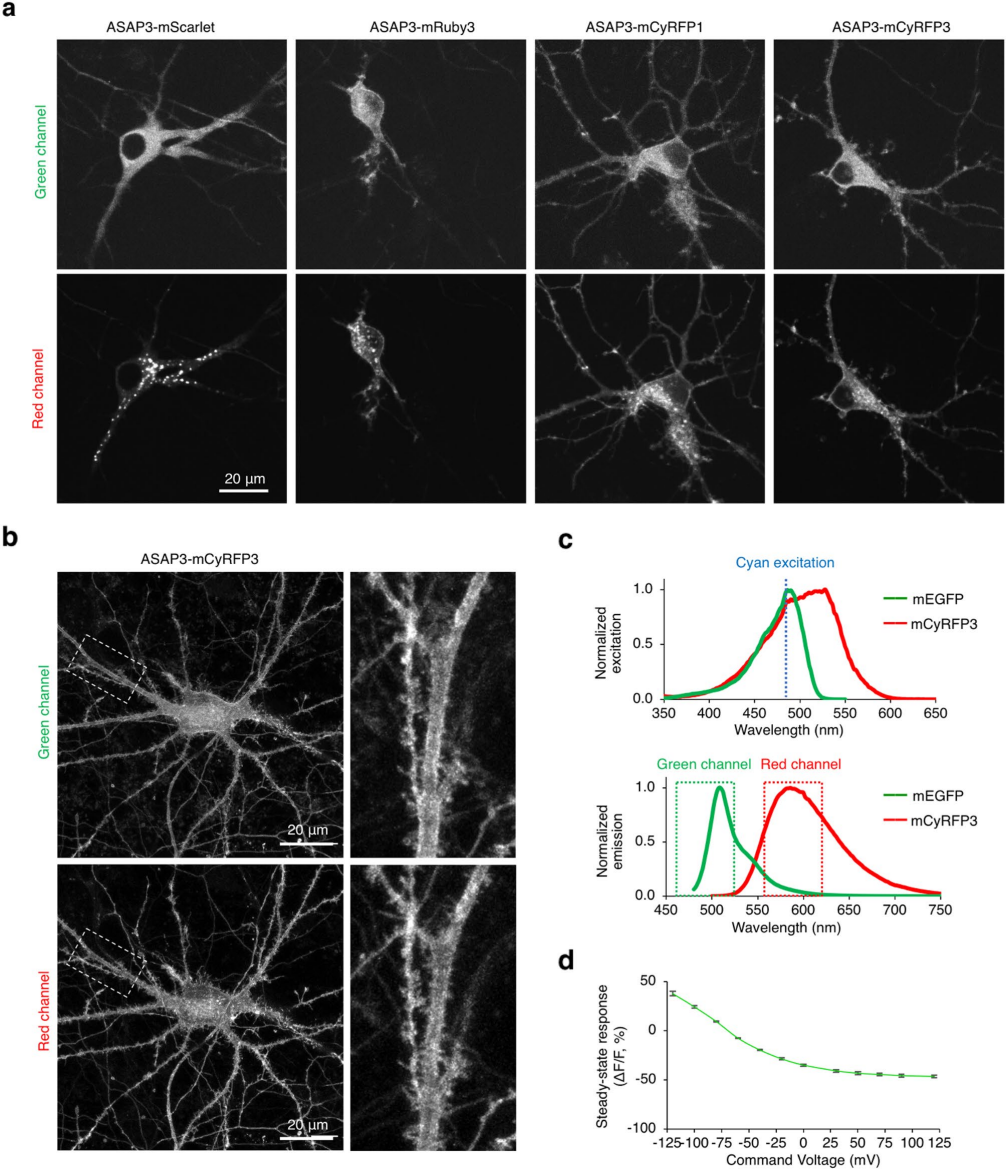
Characterization of ASAP3-mCyRFP3 (ASAP3-R3). (**a**) Representative images of ASAP3 fusions to various RFPs expressed in primary rat hippocampal neurons acquired on a spinning-disk confocal microscope using Improvision Volocity 6.0 and processed in NIH Fiji 2.1. (**b**) Laser-scanning confocal images of ASAP3-R3 in a rat hippocampal neuron. Images were acquired with Zeiss Zen Blue software and processed in Fiji 2.1. (**c**) Above, normalized excitation spectra of mCyRFP3 and mEGFP, showing a single excitation wavelength can be used. Below, normalized emission spectra of mCyRFP3 and mEGFP, showing their emissions are separable (**d**) Steady-state voltage responsivity of ASAP3-R3. Error bars represent standard error of the mean (n = 8 HEK293 cells). Graphs were made in Microsoft Excel for Mac 16.

We tested fusions of ASAP3 to mCyRFP3, mCyRFP1, mRuby3, which we had previously tested with ASAP1, and the recently developed mScarlet, which is non-perturbing in fusions with a variety of cellular proteins^15^. ASAP3-mCyRFP3 (ASAP3-R3) showed the clearest membrane localization in dendrites and the smallest amount of cell body accumulations, followed by mCyRFP1, then mRuby3, and finally mScarlet, which completely lacked membrane expression and whose cell body accumulations were especially large (Fig. 3a). We also compared ASAP3-R3 to ASAP3 fused to mCyRFP2, which was developed during the course of this work. ASAP3-mCyRFP2 showed more accumulation of red fluorescence in the cell body and less ASAP3 signal at the membrane, consistent with trapping in secretory vesicles (Supplementary Fig. 4). In neurons expressing ASAP3-R3, high-magnification confocal microscopy confirmed tight correspondence between the green signal of ASAP3 and the red signal of mCyRFP3 (Fig. 3b).

Conveniently, both fluorophores of ASAP3-R3 can be excited by the same wavelength, while their emissions are easily separable (Fig. 3c). The steady-state voltage responsivity of ASAP3-R3 in the green channel upon depolarization from − 70 mV to 30 mV, measured as the change in fluorescence intensity relative to that at the–70 mV baseline condition (∆F/F_0_), was − 41 ± 1% (mean ± standard error of the mean in eight HEK293 cells) (Fig. 3d). The slightly lower response amplitude compared to ASAP3 (− 51 ± 1%)^5^ may be due to minor contamination by voltage-insensitive mCyRFP3 fluorescence in the green emission channel (Fig. 3c).

Cardiomyocytes (CMs) derived from induced pluripotent stem cells (iPSCs) enable the study of patient-specific mechanisms in cardiovascular diseases^16^. A key functional readout of iPSC-CMs is the pattern of spontaneous cardiac APs, which drive contraction and whose appearance indicates the maturation of ion homeostasis and channel properties. Recording APs is also useful for studying signaling pathways leading to lineage specification of CM subtypes^17^. The gold standard for characterizing APs in iPSC-CMs is patch-clamp electrophysiology, but this method is low-throughput and destructive. An alternative method for AP measurements is to use fluorescent GEVIs, but GEVIs preferentially would be ratiometric so that changes in membrane potential are self-normalized and can be discriminated from motion-induced changes in fluorescence.

**Figure 4:**
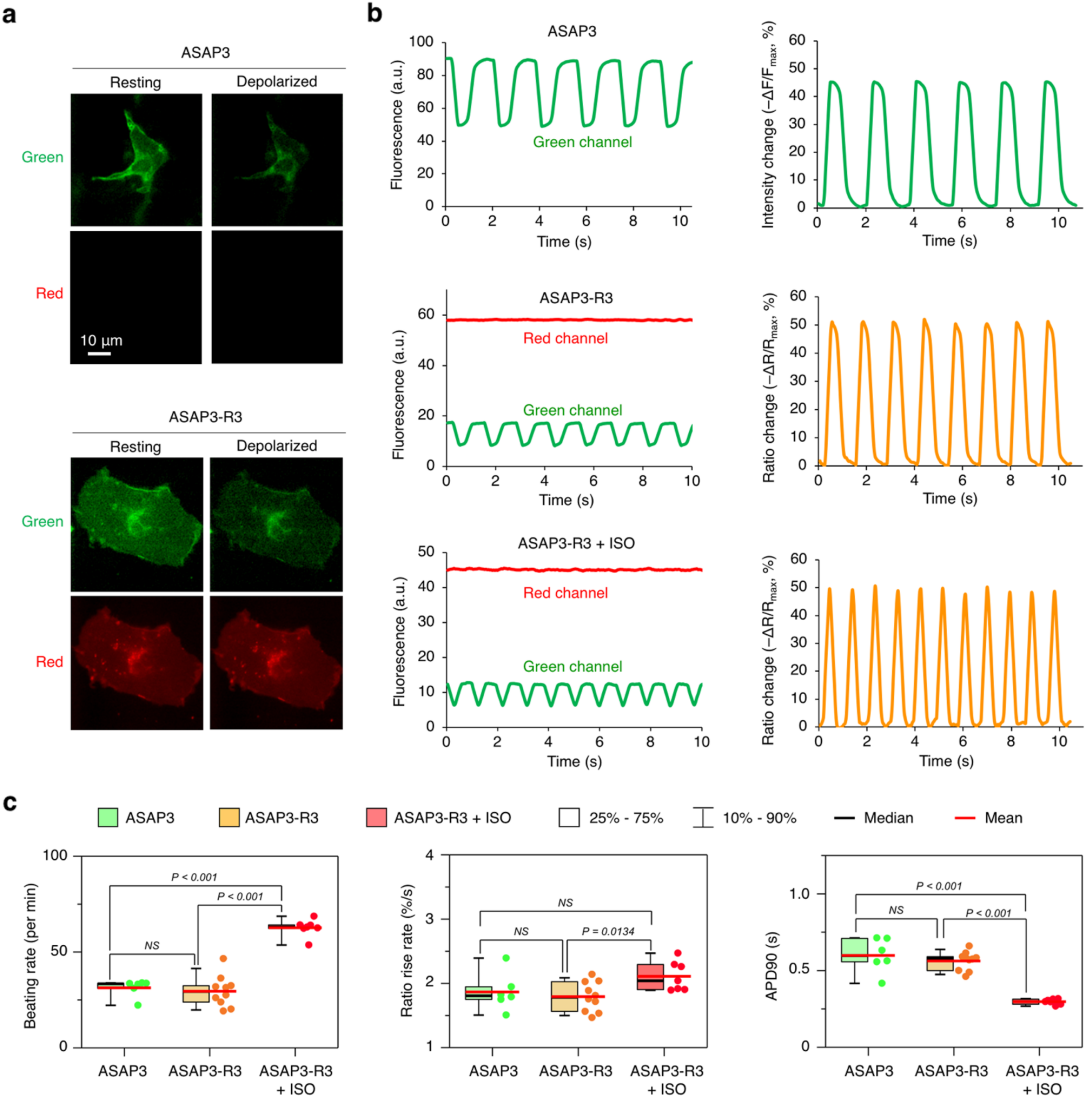
ASAP3-R3 performance in cardiomyocytes. (**a**) Representative induced pluripotent stem cell derived cardiomyocytes (iPSC-CMs) expressing ASAP3 or ASAP3-R3. Epifluorescence images were acquired using Hamamatsu HCImage software and color channels assigned in NIH Fiji 2.1. (**b**) Representative single-trial ∆R/R or ∆F/F responses to spontaneous cardiac APs of cells expressing ASAP3, expressing ASAP3-R3, or expressing ASAP3-R3 and treated with isoproterenol (ISO). Graphs were generated in Microsoft Excel for Mac 16. (**c**) Characterization of traces obtained from ASAP3 (n = 6), ASAP3-R3 (n = 10), or ASAP3-R3 + ISO (n = 7). Graphing and statistical analyses were performed in GraphPad Prism 7.

We tested the utility of ASAP3-R3 in reporting APs of iPSC-CMs. ASAP3-R3 fluorescence from beating iPSC-CMs was excited using blue light, then green and red channels were recorded by a single CMOS camera using an emission beam splitter. We found some apparently non-motile iPSC-CMs were firing APs, as ASAP3-R3 showed rhythmic decreases in fluorescence in the green channel and no change in the red channel (Fig. 4a). At the peak of the action potential (most depolarized timepoint), ASAP3-R3 showed a change in the green/red ratio relative to its maximum (most polarized) value (∆R/R_max_) of − 46.6 ± 4.8%. This was similar to the change in green fluorescence relative to maximum fluorescence (∆F/F_max_) of − 47.8 ± 5.21% for ASAP3 in spontaneously beating iPSC-CMs (Fig. 4b), demonstrating the ASAP3-R3 GEVI is fully functional. Photobleaching was not observed in the recording period (Fig. 4b).

We also tested if ASAP3-R3 can faithfully report drug induced changes of APs in iPSC-CMs. Isoproterenol (ISO) activates β-adrenergic receptor signaling in CMs to increase heart rate. ASAP3-R3 showed that ISO treatment of iPSC-CMs also increased the spontaneous contraction rate, from ~ 30 beats per minute (bpm) to > 60 bpm (Fig. 4b,c). The rise rate, which should correlate with the up-stroke velocity in patch clamp measurements, was also significantly increased (Fig. 4c). As a result, the AP duration to 90% repolarization (APD90) was significantly shorter in the ISO treated group (Fig. 4c).

**Figure 5:**
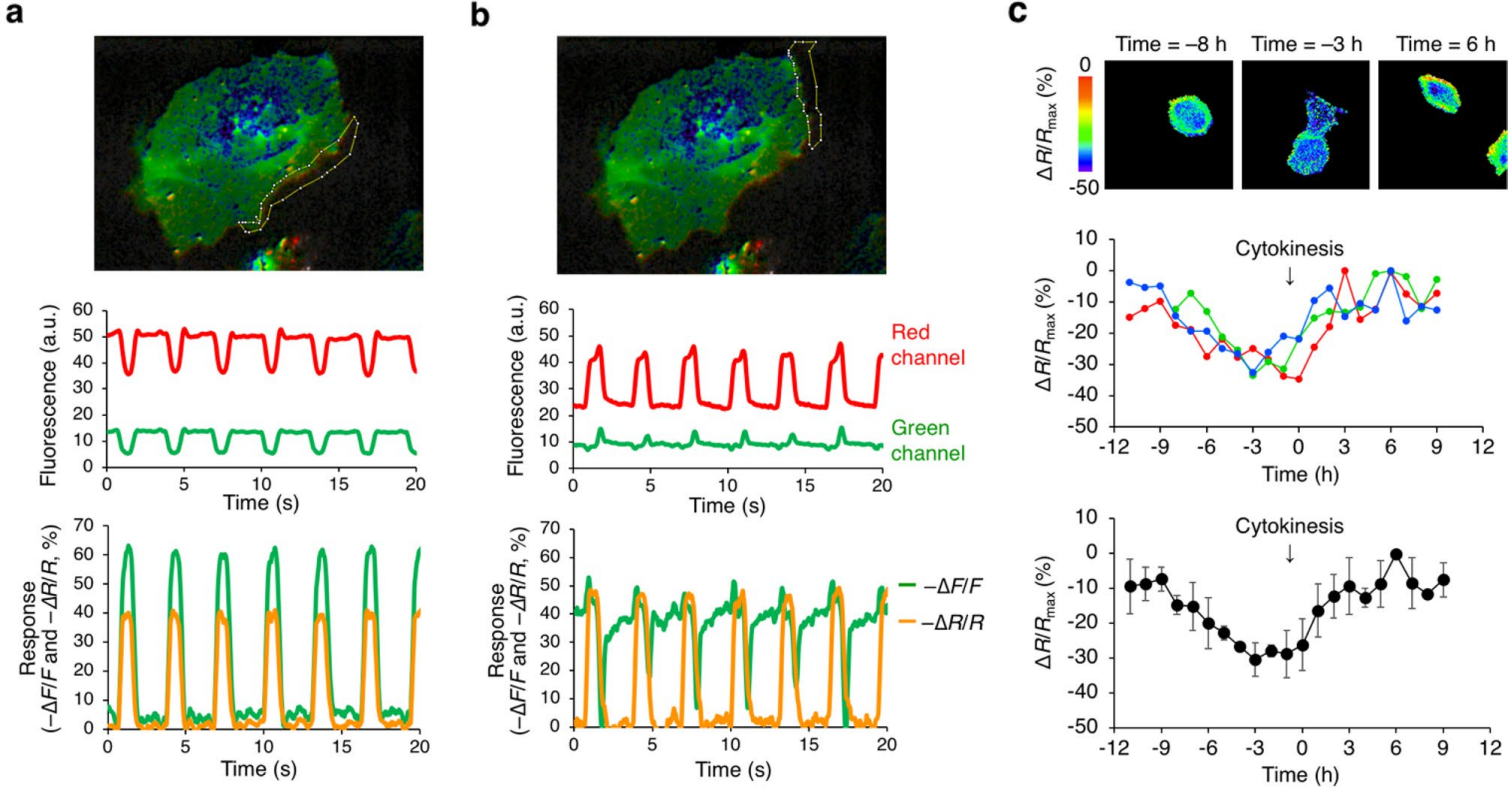
ASAP3-R3 accurately reports voltage in motile cells and detects voltage fluctuations during the cell cycle. (**a**) Top, one segment of membrane in a beating CM was analyzed. Middle, raw traces of green and red signals in the selected region. Red trace indicates significant cell movement in and out of selected region detected by the voltage-independent mCyRFP. Bottom, relative change in green fluorescence alone (− ∆F/F) and the relative change in the green/red ratio (− ∆R/R). While green intensity changes are larger than those observed previously in non-contracting CMs due to these motion artifacts, ratio changes are similar in magnitude to those observed previously. (**b**) Similar analysis on a different membrane segment of the same cell. Here, the green fluorescence changes are opposite in direction from that expected due to movement, which is detected in the red channel. While green intensity traces do not resemble AP waveforms, ratio traces are indistinguishable from AP waveforms in non-motile CMs and similar in shape to those in (**a**). Epifluorescence images were acquired using Hamamatsu HCImage software and color channels assigned and overlaid in NIH Fiji 2.1. Graphs were generated in Microsoft Excel for Mac 16. (**c**) Top, images of the ASAP3-R3 green/red ratio at different time points relative to cytokinesis in a HeLa cell. Middle, individual green/red ratio time courses of three HeLa cells during cell division. The cell in the images is represented by the blue trace. Cytokinesis occurs immediately before the 0-h time point. Below, mean green/red ratios of the three cells. Error bars represent standard deviation. Epifluorescence images were acquired using Hamamatsu HCImage software and pseudocoloring with a ratiometric lookup table was performed in NIH Fiji 2.1. Graphs were generated in Microsoft Excel for Mac 16.

We next demonstrated how ASAP3-R3 can be used to correct for motion artifacts in moving cells. Different edges of a contracting iPSC-CM can exhibited disparate responses in green intensity (Fig. 5a,b). On one edge, green signal fluctuations were larger than those in non-contracting CMs (Fig. 5a). On another edge, the green signal was inverted from the expected response and did not resemble AP waveforms (Fig. 5b). The voltage-insensitive red channel showed different rhythmic fluctuations at the two edges, indicating different patterns of membrane movement. Thus, the disparate green fluorescence responses from the same iPSC-CM were due to different contributions of membrane movement to green fluorescence. Indeed, when the green/red ratio was tracked instead, different portions of the membrane responded in the same direction and magnitude as in non-contracting iPSC-CMs (Fig. 5a,b). The ratio changes in different membrane regions also resembled each other in waveform shape even though the individual channels did not (Fig. 5a,b).

Finally, using ASAP3-R3, we demonstrated regulation of the membrane potential during the cell cycle. Potassium and chloride channel activities are regulated by cell cycle phase^18^, and electrode measurements have found fluctations in the transmembrane potential during cell cycle progression^19,20^. Interestingly, manipulations of channel function or extracellular ion concentrations suggest a role for transmembrane voltage in the timing of cell cycle transitions^18^. However, direct electrical measurements across the cell cycle have only been reported as single-timepoint measurements in non-motile cell types. We asked whether we could observe transmembrane voltage in a motile cell type throughout an entire cell cycle. HeLa cells demonstrate pronounced cellular motility, which would make repeated membrane voltage measurements of a single cell throughout the cell cycle difficult. The morphological dynamism of HeLa cells would also prevent accurate voltage readings from a non-ratiometric GEVI.

We thus expressed ASAP3-R3 in HeLa cells and visualized the green/red ratio over time. We observed a ~ 35% change in green/red ratios throughout the cell cycle, with the lowest ratio value (representing depolarization) in mitosis prior to cytokinesis (Fig. 5c). The highest green/red ratios (representing hyperpolarization) occurs 6–9 h following cytokinesis, which corresponds to S-phase in HeLa cells^13^. The timing of these voltage changes are consistent with previous reports using intracellular electrodes on non-motile cell types^19,20^. Thus, using ASAP3-R3, we were able to detect membrane voltage changes throughout the cell cycle in moving cells, finding that HeLa cells are most depolarized during mitosis and most polarized during S-phase.

Our results demonstrate the utility of structure-guided multi-site saturation mutagenesis for fluorescent protein improvement. Obtaining mCyRFP3 would have been exceedingly difficult by random mutagenesis. mCyRFP3 differs by mCyRFP1 by three nucleotides within a 700 bp gene, so in a library bearing 3 random mutations per clone without duplicates, this combination would have occurred once in 700 choose 3 × 4^3^, or 3.6 × 10^9^ mutants. In reality, the library would need to be at least an order of magnitude larger to account for Poisson distributions of mutation combinations. If the ideal combination of amino acids had required six nucleotide changes, then it would have arisen in a perfectly distributed library of six-nucleotide mutants once per 6.6 × 10^17^ clones. By identifying two sites on the crystal structure, we only needed to screen for 1024 possible combinations using an NNS codon at each site (32 × 32). Thus, hypothesis-driven structure guided mutagenesis can identify beneficial multi-site mutants that would have been unobtainable using random mutagenesis. Similar methods have been used to produce functional improvements in a variety of RFPs and in ASAP3 itself^5,7,10,13,21,22^.

Indeed, our observation that coordinated mutations at positions 148 and 161 produced more monomeric and brighter variants of multiple RFPs suggests a general strategy of monomerization in which positions interacting across a dimer interface are mutated to all possible combinations. While this may seem obvious in retrospect, this does not appear to be a standard step in the monomerization workflow for fluorescent proteins. mCyRFP1 is derived from TagRFP, which in turn is a weakly dimeric mutant of the natural tetrameric RFP eqFP578. To our knowledge, positions 148 and 161, which are distant from each other on the surface of a single monomer, have not been previously mutated in combination and to saturation in any RFPs derived from TagRFP.

As mCyRFP3 is more monomeric in vitro than mCyRFP1, it is not surprising that it would be less perturbing to ASAP3 membrane localization. However, in the OSER assay, fusions of mCyRFP1 and mCyRFP3 to CytERM showed similar propensities to form ER whorls. In addition, although FusionRed and mScarlet are less perturbing in the OSER assay than mCyRFP3, they performed worse when fused to ASAP3. These observations demonstrate that the OSER assay only tests the performance of fluorescent proteins fused to one cellular protein, and may not necessarily predict performance of fusions with any other protein. One possible complicating factor in the OSER assay is protein expression level; fusions with less stable fluorescent proteins may accumulate to lower concentrations and thereby create whorls in a smaller percentage of cells.

In summary, we have created a cyan-excitable red fluorescent protein, mCyRFP3, with improved brightness and monomericity. Among RFPs, mCyRFP3 performs especially well as a fusion tag for the ASAP3 voltage indicator. The resulting ASAP3-R3 enables voltage imaging with an internal reference channel for motion correction, as we demonstrate by imaging transmembrane voltage changes during cardiomyocyte contraction and during the cell cycle. Compared to voltage indicator dyes, ASAP3-R3 has the advantage of being genetically encoded, allowing for stable expression and targeting to specific cell types. We expect that ASAP3-R3 will also be useful for imaging voltage in the brains of living animals, where the mCyRFP3 reference channel can allow for correction of motion artifacts in the voltage-modulated ASAP3 signal.

## Methods

### Mutagenesis and screening of libraries

Plasmids were constructed using standard molecular biology methods including polymerase chain reaction (PCR) and In-fusion cloning (Clontech). Mutations for specific residues were introduced by overlap-extension PCR. All cloning junctions and PCR products were sequence verified. Mutants were expressed and screened in constitutively active bacterial expression vector pNCS (Allele Biotech). Plasmids were transformed into chemically competent XL-10 Gold (Agilent), and colonies were grown on LB agar plates at 37 °C for 16–20 h and at room temperature for an additional 20–24 h. For each round of mutagenesis, the number of colonies screened was tenfold the expected library diversity to ensure full coverage. Colonies expressing mCyRFP3 variants were screened for transmitted color by eye and for fluorescence in a Fluorochem Q imaging enclosure (Alpha Innotech) with an 475/42-nm excitation filter and an 699/62-nm emission filter.

### Protein production and characterization

For spectral characterization, bacterial pellets were lysed in B-PER II (Pierce) and hexahistidine-tagged proteins were purified with HisPur Cobalt Resin (Pierce). Proteins were desalted into phosphate-buffered saline (PBS) pH 7.4 using Econo-Pac 10DG gravity flow chromatography columns (Bio-Rad). Absorbance, excitation spectra, and emission spectra were measured with Safire2 or Infinite M1000 Pro plate readers (TECAN). Extinction coefficients were calculated using the base-denaturation method^21^. Quantum yields for mCyRFP3 were determined in PBS at pH 7.4 by exciting with 475- to 485-nm light and integrating emission from 500 to 800 nm, corrected for detector sensitivity. mCyRFP1 was used as the quantum yield standard. In vitro photobleaching measurements were performed in PBS droplets under mineral oil on an IX81 inverted microscope with a 40 × 1.15-numerical aperture (NA) water-immersion objective, an X-Cite 120-W metal halide lamp (Lumen Dynamics) at 100% neutral density, a 485/10-nm excitation filter (Omega), and an Orca ER camera (Hamamatsu) controlled by Micro-Manager software^23^. Images were acquired every 1 s under continuous illumination. Times were scaled to produce photon output rates of 1000 per molecule per s as previously described^24^. pH titration was performed using a series of buffers (1 M HOAc, 1 M NaOAc, 5 M NaCl for pH 3–5.5; 100 mM KH_2_PO_4,_ 100 mM K_2_HPO_4_ for pH 6–8; 100 mM glycine for pH 9.5–10). HCl or NaOH were used to adjust the pH. 5 μL of purified protein was diluted in 145 μL buffer with different pH values, and the fluorescence brightness was measured. Size exclusion chromatography was performed with a Superdex 200 30/100 GL column (GE Healthcare). 100 μL of 10–100 μM purified proteins were loaded and eluted at a flow rate of 0.5 mL/min. Protein elution was monitored by absorbance at 280 nm.

### Brightness comparison of fluorescent proteins in mammalian cells

HeLa (ATCC) and HEK293A (GE Dharmacon, Fischer Scientific) cells were grown on glass-bottom dishes (Cellvis) in high-glucose Dulbecco's Modified Eagle Medium (DMEM, Hyclone) supplemented with 10% fetal bovine serum (Gemini), 2 mM glutamine (Gemini), 100 U/mL penicillin and 100 μg/mL streptomycin (Gemini) to 70–80% confluency, then transfected using Lipofectamine 2000 (Thermo Fisher) with a EGFP-P2A-RFP-CAAX construct, where RFP was CyRFP1, mCyRFP1, or mCyRFP3. EGFP served to normalize for transfection efficiency and cell number. One day post-transfection, approximately 10^5^ cells were replated in a 96-well plate and emission spectra from 500 to 800 nm, upon excitation at 480 nm with a bandwidth of 10 nm, were obtained in an Infinite M1000 Pro microplate reader (Tecan). As EGFP emission is negligible at peak RFP emission and vice versa, the EGFP-normalized RFP brightness was simply calculated as the ratio of RFP and EGFP peak values.

### Microscopy of fusion proteins in non-neuronal cells

mCyRFP3 fusions were cloned into pLL3.7 m, a modified form of pLL3.7, using standard molecular biology methods. Fusions were made to human calnexin (NM_001746.3), Lifeact, mouse mannosidase II (NM_008549.2), human laminB1 (NM_005573.2), human pyruvate dehydrogenase (NM_000284), chicken paxillin (NM_204984.1), human histone H2B (NM_021058.3), rat connexin-43 (NM_012326.2), and human α-tubulin (NM_006082). All sequences were gifts of M. Davidson (Florida State University). mCyRFP3 fused to the CAAX membrane-localization signals were subcloned into pcDNA3. HeLa cells were grown and transfected as above, then imaged 24–48 h later in FluoroBrite DMEM with B-27. mCyRFP3 fusions were imaged on an Axio Observer microscope with a 63 × 1.3-NA oil-immersion objective (Zeiss) equipped with an UltraVIEW spinning-disc confocal unit (Perkin-Elmer). Excitation was provided by a 488-nm laser excitation, and emission was collected through a 615/70-nm filter with an C9100-50 EMCCD camera (Hamamatsu). Images were acquired using Improvision Volocity 6.0. Maximal intensity projections of optical sections were generated in the ImageJ program^25^.

### Organized smooth endoplasmic reticulum (OSER) assay

mCyRFP3, EGFP, and mCardinal were fused to the C-terminus of the signal-anchor transmembrane domain of cytochrome P450 (amino acids 1–29, CytERM) in a pcDNA3 vector using standard molecular biology methods. HeLa cells were grown and transfected as above, then imaged 18 h post-transfection in FluoroBrite DMEM with B-27 on the Axio Observer microscope with a 63 × 1.3-NA objective (Zeiss) equipped with an UltraView spinning-disc confocal unit (Perkin-Elmer). The percentage of cells with visible whorls were counted, excluding cells with unusually bright expression. At least 150 cells were counted per dish, and three technical replicates using three dishes were performed. CytERM-EGFP and CytERM-mCardinal served as references for comparison to previously reported data^26^.

### Neuronal cell culture, transfection, and imaging

All procedures were approved and carried out in compliance with the Stanford University Administrative Panel on Laboratory Animal Care, and all experiments were performed in accordance with relevant guidelines and regulations. Hippocampal neurons were extracted from embryonic day 18 Sprague Dawley rat embryos by dissociation in HBSS supplemented with 10 mM d-glucose and 10 mM HEPES pH 7.2 (Thermo Life Sciences). Digestion was performed in RPMI media containing 20 U/mL papain (Worthington Biochemical LS003119) and 0.005% DNAase I, at 37 °C for 15 min. Dissociated neurons were then plated in Neurobasal with 10% FBS, 2 mM GlutaMAX, and 2% B27 (Thermo Fisher) at a density of 4 × 10^6^ cells/cm^2^ in a 12-well glass-bottom plate, pre-coated overnight with 0.1 mg/mL > 300-kDa poly-d-lysine hydrobromide (Sigma). 6–8 h later media was replaced with Neurobasal with 1% FBS, 2 mM GlutaMAX, and B27, with refreshing media every 3–4 days. Neurons were then transfected at 9–11 days in vitro with Lipofectamine 2000 (Thermo Fisher). 500 ng of total DNA (100 ng ASAP3-RFP and 400 ng pcDNA3) and 1.5 µL of Lipofectamine 2000 transfection reagent was used for each well. The culture medium from each well was first collected and stored at 37 °C and 5% CO_2_ and temporarily replaced with Neurobasal with 2 mM GlutaMAX. Neurons were imaged 3 days post-transfection on either an inverted fluorescence microscope (Olympus IX81 or Zeiss Axiovert 200M) with a 20 × 0.75-NA objective, a metal-halide arc lamp (Exfo), and an Flash 4.0 sCMOS camera (Hamamatsu) controlled with MicroManager 1.4 software, or on an Ultraview spinning disk confocal system running Volocity software (Perkin Elmer) with a 20 × 0.75-NA air objective (Zeiss) and a ImagEM C9100-13 camera (Hamamatus). Excitation was provided by a 488-nm laser and emission was collected with 527/55-nm and 610/70-nm emission filters. Alternatively, images were captured on a LSM 880 confocal microscope with a 40 × 1.3-NA oil-immersion objective running Zen Blue software (Zeiss). Excitation was provided by a 488-nm laser and emission was collected simultaneously at 493–566 nm and 565–656 nm. Maximal intensity projections of optical sections were generated in Fiji 2.1.

### Differentiation, culture, transfection, and voltage imaging of human iPSCs derived cardiomyocytes

Stem cell protocols were approved from the Stanford University Human Subjects Research Institution Review Board. Human iPSC lines were maintained in 5% CO_2_ environment in Essential 8 Medium (Thermo Fisher). For cardiomyocyte differentiation, iPSCs were switched to insulin-free RPMI + B27, where they were treated with 6 µM CHIR99021 (Selleckchem) for 2 days, recovered for 1 day, and 5 µM IWR-1 (Sigma) for 2 days, recovered for another 2 days, and finally switched to RPMI + B27 plus insulin medium. After beating CMs were observed around day 9–11 of differentiation, iPSC-CMs were reseeded and purified with glucose-free RPMI + B27 medium for 2 rounds. The purity and quality of the iPSC-CMs were verified by FACS with TNNT2 and immune-staining of TNNT2 and α-actinin. The iPSC-CMs were maintained in RPMI + B27 medium until day 30 and were dissociated with TrypLE Select Enzyme (Thermo Fisher) and reseeded in glass-bottom dishes at a concentration of 50 K cell per square centimeter. Briefly, 1 µg of plasmid ASAP3 and ASAP3-R3 were added into the solution of GeneJammer (Agilent) transfection reagent in Opti-MEM (3 µL in 200 µL), and incubated for 30 min at RT. Then the mixtures were added directly into the cell culture and incubated for 12 h. The cell cultures were allowed to incubate for an additional 3–4 days for optimal transfection results. Cells were then imaged at 20 Hz on an Axiovert 200 M inverted microscope (Zeiss) with a 40 × 1.2-NA C-Apochromat water-immersion objective and equipped with a stage-top incubator (LCI). Excitation from a 150-W xenon arc lamp (Zeiss) at 100% neutral density was passed through a 480/30 nm excitation filter (Chroma). Emissions were simultaneously collected in green (530/30 nm) and red (630/50) channels using a DualView image splitter (Mag Biosystems). Images were acquired on an ORCA-Flash4.0 V2 C11440-22CU scientific CMOS camera with a HCImage software (Hamamatsu). At least 10 cells were selected for recording. Fluorescence intensity in cellular regions of interest or a background region were measured in NIH Fiji 2.1. Mean background intensity was subtracted from cellular regions of interest. Graphs of fluorescence traces were generated in Excel for Mac 16 (Microsoft). Statistics and graphing of iPSC-CM activity parameters were performed in Prism 7 (GraphPad).

## Supplementary Information

Supplementary Information.
