# Supplementary material for: A red fluorescent protein with improved monomericity enables ratiometric voltage imaging with ASAP3
Source: Sci Rep. 2022 Mar 7;12:3678. doi: 10.1038/s41598-022-07313-1 (PMC8901740; doi:10.1038/s41598-022-07313-1)
Supplement: Supplementary file 1 — Supplementary Information. [file 41598_2022_7313_MOESM1_ESM.pdf]

A red fluorescent protein with improved monomericity enables ratiometric voltage imaging with ASAP3

Benjamin B. Kim, Haodi Wu, Yukun A. Hao, Michael Pan, Mariya Chavarha, Yufeng Zhao, Michael Westberg, François St-Pierre, Joseph C. Wu, Michael Z. Lin

SUPPLEMENTARY MATERIALS

Supplementary Note

We hypothesized that combinatorial mutagenesis of Tyr-148 and Ala-161 could also improve monomericity and maturation of other TagRFP derivatives. The TagRFP derivative mMaroon1 features far-red emission that provides an additional channel distinct from orange fluorescent proteins[13]. However, mMaroon1 is only weakly monomeric, migrating on native polyacrylamide gel electrophoresis as a monomer but eluting as a dimer in size-exclusion chromatography (SEC) at 10 μM (**Supplementary Fig. 2a**). We thus performed combinatorial saturation mutagenesis of positions 148 and 161 in mMaroon1 to improve its monomericity and maturation. Indeed, we identified one mutant, mMaroon1 Y148T A161G, that was as bright as mMaroon1 in bacteria and exhibited improved monomericity, eluting in SEC as a dimer-monomer mixture at 10 μM (**Supplementary Fig. 2a**).

To further enhance monomericity, we explored removal of the 20-residue C-terminal tail, which engages in an dimerizing interaction in crystal structures of Neptune1, the most recent evolutionary predecessor to mMaroon1 for which a crystal structure exists[22]. In SEC, mMaroon1 ΔCT at 10 μM eluted in a broad peak overlapping with the dimeric standard (**Supplementary Fig. 2b**). Interestingly combining Y148T A161G mutations and tail deletion created a protein that was monomeric at 100 μM (**Supplementary Fig. 2b**). However, mMaroon1 Y148T A161G ΔCT was much dimmer in bacteria than mMaroon1 (**Supplementary Fig. 2c)**. This was despite similar chromophore maturation efficiency as a fraction of purified protein (75% for mMaroon1 Y148T A161G ΔCT vs. 79% for mMaroon1), suggesting protein instability rather than slow maturation as the cause of low fluorescence. Random mutagenesis of mMaroon1 Y148T A161G ΔCT identified an E175K mutant as maturing well in bacteria **(Supplementary Fig. 2c)** while maintaining monomericity at 100 μM **(Supplementary Fig. 2d)**. This protein, designated mMaroon2, exhibited peak absorbance and excitation at 604–606 nm (**Supplementary Fig. 2e,f**), a slightly red-shifted emission peak at 658 nm **(Supplementary Fig. 2f)**, and an extinction coefficient of 81 mM^{-1} cm^{-1} **(Table 1)**. Excitation by cyan light revealed a smaller residual green component compared to mMaroon1 **(Supplementary Fig. 2g)**. mMaroon2 displayed a pK_a of 6.1 **(Supplementary Fig. 2h)**, a maturation half-time of t = 8 min **(Supplementary Fig. 2i)**, and a normalized photobleaching half-time of 90 s **(Supplementary Fig. 2j)**.

We characterized the performance of mMaroon2 in mammalian cells. A fusion of histone 2B with mMaroon2 did not interfere with mitosis **(Supplementary Fig. 3a)** and fusions of various subcellular proteins to mMaroon2 were properly localized **(Supplementary Fig. 3b)**. We compared the brightness of cells expressing the parent dimer mMaroon1, mMaroon1 Y148T A161G ΔCT, and mMaroon2 from a bicistronic construct co-expressing mTurquoise2, using mTurqouise2 fluorescence to normalize for any differences in transfection efficiency or mRNA stability. In HEK293A cells, mMaroon2 was as bright as mMaroon1 and five-fold brighter than mMaroon1 Y148T A161G ΔCT **(Supplementary Fig. 3c)**. In HeLa cells, mMaroon2 was almost two-fold brighter than mMaroon1 and eight-fold brighter than mMaroon1 Y148T A161G ΔCT **(Supplementary Fig. 3c)**.

Supplementary Table 1. Engineering of mMaroon2

	Peak excitation (nm)	Peak EC ($mM^{-1}cm^{-1}$)	Peak emission (nm)	QY	Brightness ($M^{-1}cm^{-1}$)	Multimericity (at 10 μM)
mMaroon1	608	80	654	0.11	8.8	Dimer
mMaroon1 Y148T A161G	606	90	656	0.10	9.0	Monomer-dimer mixture
mMaroon1 ΔCT	606	93	660	0.11	10	Monomer-dimer mixture
mMaroon1 Y148T A161G ΔCT	604	79	658	0.10	7.9	Monomer
Maroon1 Y148T A161G E175K ΔCT (mMaroon2)	604	81	658	0.10	8.1	Monomer

EC, extinction coefficient. QY, quantum yield.

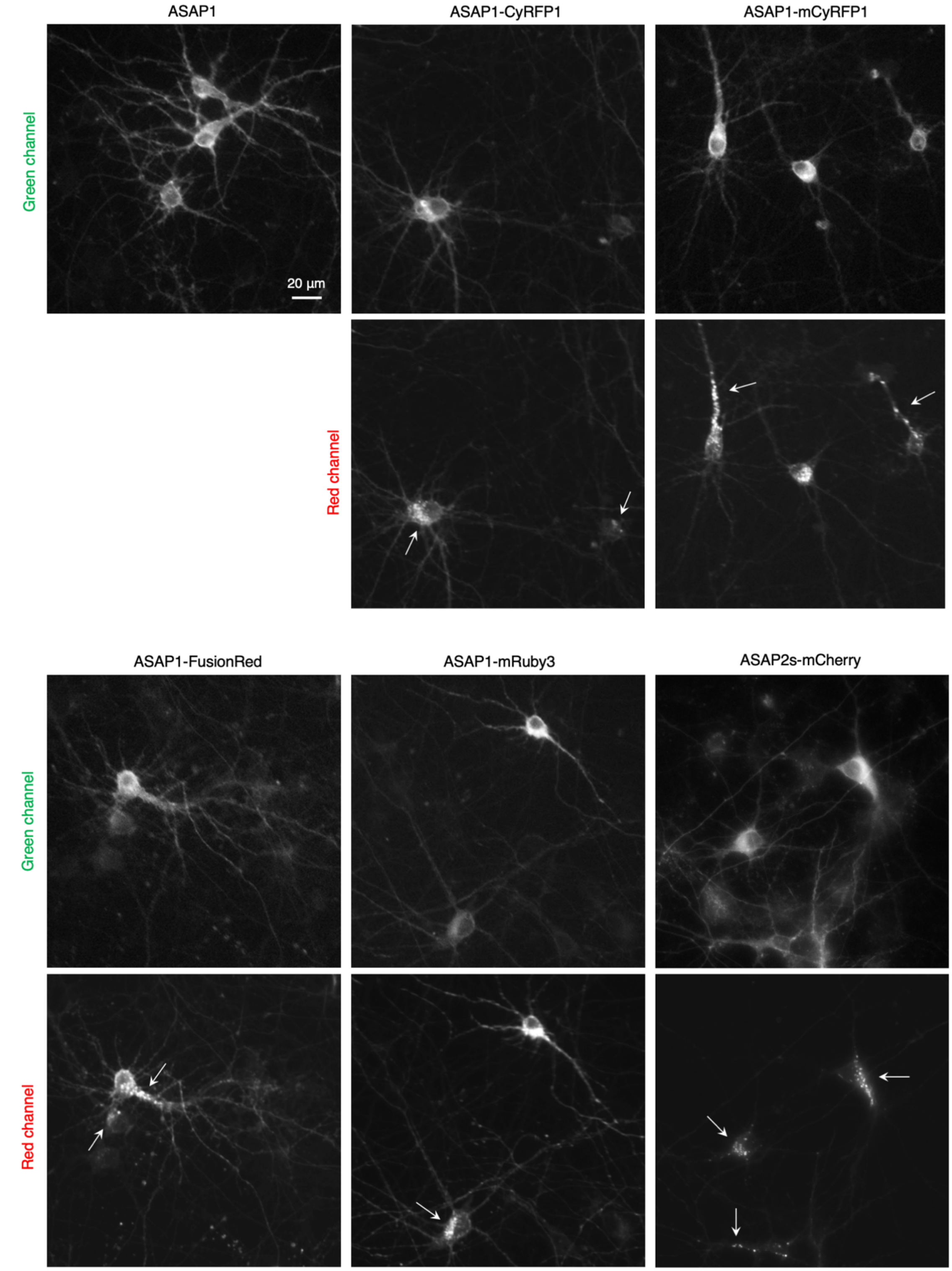

Supplementary Figure 1. Initial candidates for ratiometric ASAP expressed in rat hippocampal neurons. Arrows point to large accumulations of the RFP-tagged ASAPs in the cell body or neurites. Epifluorescence images were acquired with an sCMOS camera using MicroManager 1.4 and cropped and scaled using NIH Fiji 2.1.

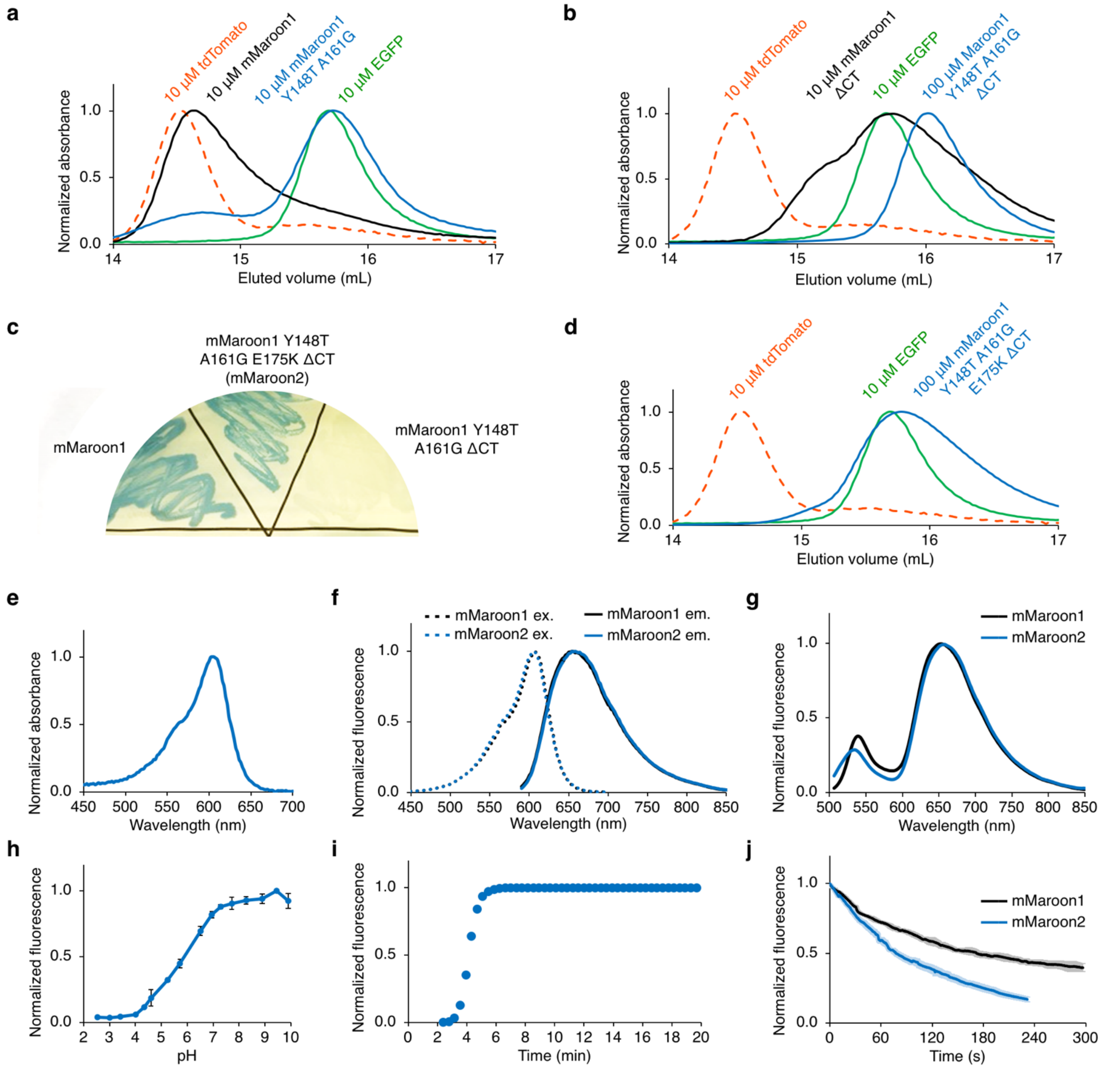

Supplementary Figure 2. Characterization of mMaroon2. (a) In SEC, mMaroon1 elutes as a dimer while mMaroon1 Y148T A161G elutes as a dimer-monomer mixture at 10 µM concentration. EGFP was used as a monomeric standard, while tdTomato was used as a dimeric standard. (b) In SEC, 10 µM mMaroon1 ΔCT elutes with a broad size distribution while 100 µM mMaroon1 Y148T A161G ΔCT elutes as a monomer. (c) Patches of bacteria expressing mMaroon1, mMaroon1 T148 G161 K175 ΔCT (mMaroon2), and mMaroon1 T148 G161 ΔCT grown for 2 d. (d) In SEC, 100 µM mMaroon1 T148 G161 K175 ΔCT (mMaroon2) elutes as a monomer. (e) Absorbance of mMaroon2. (f) Excitation and emission of mMaroon2. (g) Emission of mMaroon1 and mMaroon2 excited at 480/10 nm and collected from 500 nm to 800 nm. (h) The pKa of mMaroon2 was measured as 6.1. Error bars are s.e.m of triplicate measurements. (i) Maturation kinetics of mMaroon2. (j) Photobleaching kinetics of purified mMaroon1 or mMaroon2 with a 120-W metal-halide arc lamp through a 615/30-nm excitation filter. The time axis was adjusted for each fluorophore to simulate excitation conditions producing 1000 photons per s per molecule. Lighter shading represents standard deviation of five measurements. Graphs were generated in Microsoft Excel for Mac 16.

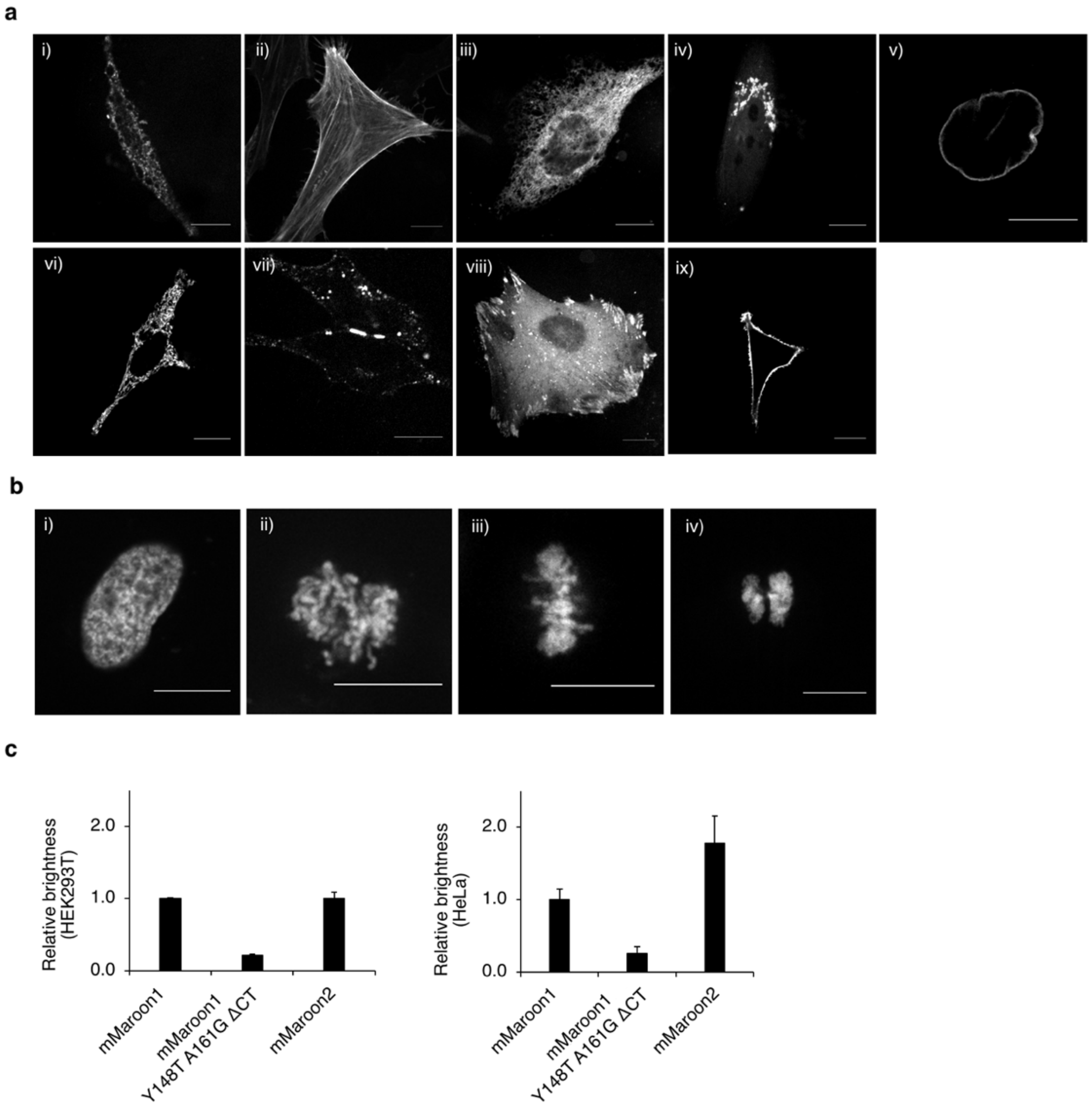

Supplementary Figure 3: Performance of mMaroon2 Fusions. (a) HeLa cells expressing mMaroon2 fused to various domains. For each fusion, the original of the fusion partner and its normal subcellular location are indicated in parentheses. (i) mMaroon2-2aa-tubulin (human, microtubules), (ii) mMaroon2-7aa-actin (human, actin cytoskeleton), (iii) Calnexin-14aa-mMaroon2 (human, endoplasmic reticulum), (iv) mannosidaseII-10aa-mMaroon2 (mouse, Golgi complex), (v) mMaroon2-10aa-lamin B1 (human, nuclear envelope) (vi) PDHA-10aa-mMaroon2 (human, mitochondrial pyruvate dehydrogenase), (vii) connexin43-7aa-mMaroon2 (rat, cell-cell adhesion junctions), (viii) paxillin-22aa-mMaroon2 (chicken, focal adhesions), (ix). mMaroon2-2aa-CAAX. Scale bar, 10 µm. (b) mMaroon2-10aa-H2B (human, nucleosomes) in (i) interphase, (ii) prophase, (iii) metaphase, (iv) anaphase. Spinning-disk confocal image stacks were acquired using Improvision Volocity 6.0 and flattened and scaled in NIH Fiji 2.1. (c) Brightness comparison of mMaroon2 in HEK293A and HeLa cells expressing the bicistronic construct mTurquoise2-P2A-RFP where RFP is mMaroon1, mMaroon1 Y148T A161G ΔCT, or mMaroon2. The red fluorescence generated from each of the RFPs was divided by the fluorescence of mTurquoise2 to correct for transfection and then normalized to the value of mMaroon1. Graphs are mean ± s.e.m. from six measurements. Graphs were generated in Microsoft Excel for Mac 16.

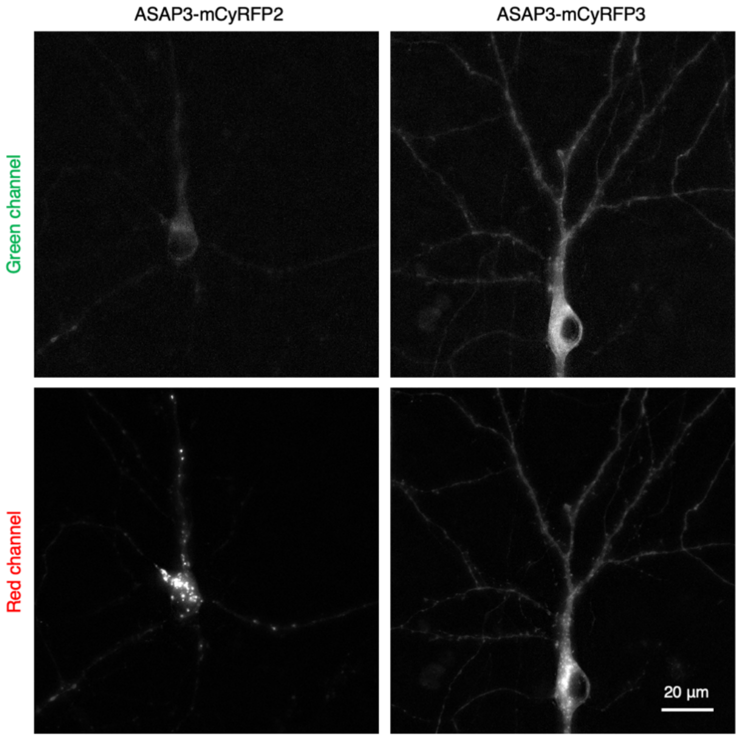

Supplementary Figure 4. Testing fusion of ASAP3 to mCyRFP2 or mCyRFP3. Representative images of ASAP3 fused to mCyRFP2 or mCyRFP3 acquired by epifluorescence are shown. Epifluorescence images were acquired on an sCMOS camera using MicroManager 1.4 and cropped and scaled using NIH Fiji 2.1.
